# Low risk of attrition among adults on antiretroviral therapy in the Rwandan national program: a retrospective cohort analysis of 6, 12, and 18 month outcomes

**DOI:** 10.1186/1471-2458-14-889

**Published:** 2014-08-29

**Authors:** Harriet Nuwagaba-Biribonwoha, Aleksandra Jakubowski, Veronicah Mugisha, Paulin Basinga, Anita Asiimwe, Denis Nash, Batya Elul

**Affiliations:** ICAP, Mailman School of Public Health, Columbia University, New York, New York USA; Department of Epidemiology, Mailman School of Public Health, Columbia University, New York, New York USA; Bill and Melinda Gates Foundation, Seattle, Washington USA; Rwanda Biomedical Center [RBC], Ministry of Health, Kigali, Rwanda; Hunter College, City University of New York, New York, New York USA

**Keywords:** Antiretroviral therapy [ART], Retention, Attrition, Mortality, Loss to follow-up, Rwanda

## Abstract

**Background:**

We report levels and determinants of attrition in Rwanda, one of the few African countries with universal ART access.

**Methods:**

We analyzed data abstracted from health facility records of a nationally representative sample of adults [≥18 years] who initiated ART 6, 12, and 18 months prior to data collection; and collected facility characteristics with a health facility assessment questionnaire. Weighted proportions and rates of attrition [loss to follow-up or death] were calculated, and patient- and health facility-level factors associated with attrition examined using Cox proportional hazard models.

**Results:**

1678 adults initiated ART 6, 12 and 18 months prior to data collection, with 1508 person-years [PY] on ART. Attrition was 6.8% [95% confidence interval [CI] 6.0-7.8]: 2.9% [2.4-3.5] recorded deaths and 3.9% [3.4-4.5] lost to follow-up. Population attrition rate was 7.5/100PY [6.1-9.3]. Adjusted hazard ratio [aHR] for attrition was 4.2 [3.0-5.7] among adults enrolled from in-patient wards [vs 2.2 [1.6-3.0] from PMTCT, ref: VCT]. Compared to adults who initiated ART 18 months earlier, aHR for adults who initiated ART 12 and 6 months earlier was 1.8 [1.3-2.5] and 1.3 [0.9-1.9] respectively. Male aHR was 1.4 [1.0-1.8]. AHR of adults enrolled at urban health facilities was 1.4 [1.1-1.8, ref: rural health facilities]. AHR for adults with CD4+ ≥200 cells/μL vs <200 cells/μL was 0.8 [0.6-1.0]; and adults attending facilities with performance-based financing since 2004–2006 [vs. 2007–2008] had aHR 0.8 [0.6-0.9].

**Conclusions:**

Attrition was low in the Rwandan national program. The above patient and facility correlates of attrition can be the focus of interventions to sustain high retention.

## Background

Millions of HIV-infected adults have initiated antiretroviral therapy [ART] in the past decade, but the overall impact of these programs can be compromised by patient attrition, including death and loss to follow-up [1–8]. Rwanda is one of a handful of African countries to have achieved universal ART access, with at least 80% of the people eligible for ART receiving it [9, 10]. ART services in Rwanda were established in 2002, and by 2012, over 100,000 HIV-infected patients had initiated ART at more than 400 health facilities [11, 12]. High levels of retention on ART were reported for the first few years of the national program [13, 14]; indeed, in a nationally representative study conducted in 2004–2005, 92% and 86% of patients who initiated ART 6 and 12 months earlier, respectively, were alive and on ART at the health facility of ART initiation [13]. Over time, the national program expanded significantly, with over a five-fold increase in the number of facilities providing ART and the number of patients initiating ART [12]. As Rwanda, continues to build sustainable ART programs, it is important to continually assess whether these high levels of retention can be sustained with program scale-up and universal ART access. We use data from a nationally representative study of adults initiating ART to report on levels and correlates of attrition.

## Methods

### Study design and data collected

**Figure 1:**
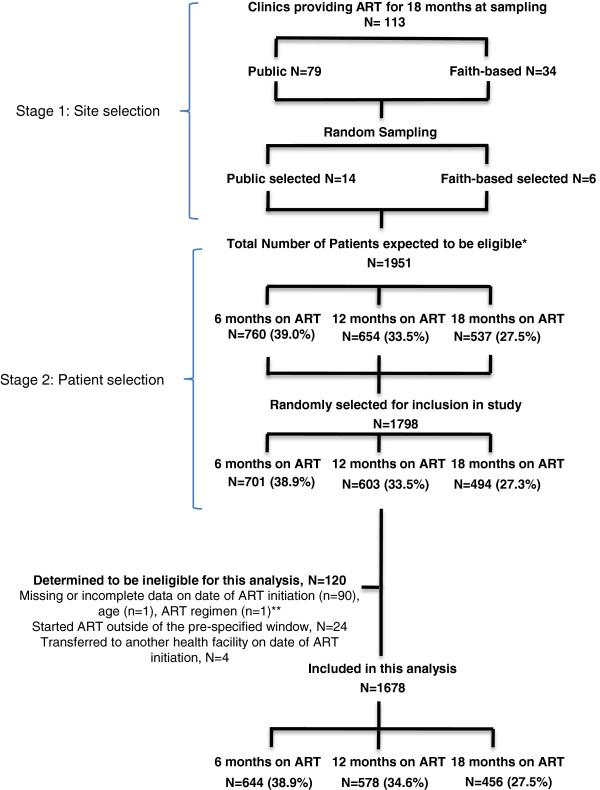
**Sampling and participant selection.** *Data obtained from TRACNet, the national monitoring and evaluation system. **Data not found in clinic records at the time of data abstraction.

The data presented here were collected between September 2008 and April 2009 as part of a nationally-representative study of adherence among adults on ART. The full methods have been described elsewhere [15], but briefly multi-stage sampling was used to select 1,798 adults who had initiated ART approximately 6, 12 and 18 months prior to data collection across 20 HIV health facilities from the total of 9,693 adults receiving ART at 113 health facilities in Rwanda at the time of sampling (Figure 1). Data from patient medical charts and pharmacy records—including demographic and clinical characteristics, dates of ART initiation and health facility visits, and patient outcomes—were abstracted by trained study personnel using a structured tool. A health facility assessment questionnaire captured information on characteristics of each of health facility including the type and location of the health facility in which HIV services were located, availability of supportive services for ART patients e.g. home visits and timing of the introduction of performance based financing. In this setting, performance based financing was a supply-side intervention that provided health facilities a payment mechanism based on fees for services (conditional on quality) to motivate health workers to increase quality health outputs. Key HIV indicators (like number of patients tested for HIV, number of patients newly initiating ART) were rewarded with financial incentives and infrastructural support. We hypothesized those facilities that had performance based financing for longer would be motivated to ensure better health outcomes among patients, including retention.

### Inclusion criteria

During the study period the national adult ART initiation criteria in Rwanda were CD4 < 200 cells/μL or WHO stage IV, with stavudine and zidovudine as the main nucleoside reverse-transcriptase inhibitors used in first line ART regimens [16]. Patients sampled for the study described above were eligible for this analysis if they were 18 years or older, and had initiated first-line ART at one of the study facilities 6, 12, and 18 months [+/− 2 months] prior to data collection or transferred into one of the study facilities within 30 days of ART initiation. We aimed to determine the population estimate of attrition by conducting secondary analysis of an existing dataset, using the entire sample. Basing on Lowrance et al’s estimates of death and lost to follow-up, we determined that a dataset of 1798 would allow detection of attrition between 5% and 7% with >90% power and 5% precision. Of the 1798 adults originally sampled, 120 were excluded as they started ART outside of the above-specified timeframe [n = 24], were missing information on the date of ART initiation [n = 90], ART regimen [n = 1], and age [n = 1], or transferred to another health facility on the same day as starting ART [n = 4], resulting in a final analytic sample of 1678 adults (Figure 1).

### Outcome

The outcome for this analysis was attrition, which included patients who were known to have died and those who were lost to follow-up [LTF]. Adults were considered LTF if they were not known to have died or transferred to another health facility and had not made a clinical or pharmacy visit in the 90 days before data abstraction. Adults were considered retained on ART if they picked up ART in the 90 days preceding data abstraction. Adults who were recorded as transferring out to another health facility were reported in a separate category, and not included in the retained or attrition outcomes.

### Statistical methods

Statistical analysis was conducted using Stata software version 12.1 [StataCorp. 2011. Stata Statistical Software: Release 12. College Station, TX: StataCorp LP]. Survey procedures, including finite population correction, were used to adjust estimates for the complex survey design. We applied sampling weights that took into account the probability of a health facility being selected from the 113 health facilities providing ART at the time of sampling and the probability of a participant being selected from all adults who initiated ART at 6, 12 and 18 months previously, and thus results presented in this paper represent population-wide estimates of attrition. Descriptive statistics were computed for patient- and health facility-level characteristics. We calculated the proportion and rates per 100 person years [PY] of attrition for the entire population and by time on ART [i.e. 6, 12 and 18 months], and the 95% confidence interval around these estimates. Adults who transferred to another health facility were censored at the date of transfer.

Patient- and health facility-level factors associated with attrition were modeled using weighted data in bivariate and multivariable Cox proportional hazard models to calculate crude and adjusted hazard ratios and 95% confidence intervals. Variables included in the model were pre-selected according to hypothesised relevance and data availability, excluding variables that were collinear or lacked heterogeneity. We aggregated adults who had initiated ART 6, 12, and 18 months prior to data collection to increase statistical power, and controlled for time since ART initiation in the multivariable model.

### Ethical considerations

The study protocol was approved by the Rwanda National Ethics Committee and the Institutional Review Board at Columbia University. This study was funded through the President’s Emergency Plan for AIDS Relief [PEPFAR] through the Centers for Disease Control and Prevention [CDC] and received Associate Director of Science [ADS] clearance. This analysis was based on data collected by abstraction from existing medical records; individual-level patient consent was waived by all approving ethical boards, and not sought.

## Results

A total of 1678 adults were included in this analysis, 644 [37.4%], 578 [33.3%], and 456 [29.3%] who had initiated ART 6, 12 and 18 months prior to data collection, respectively. The total amount of follow-up time was 1508 person years, while the average follow-up time was 11.3 months. The weighted population was 8,373, 3123 [37.3%], 2762 [33.0%], and 2488 [29.7%] patients who had initiated ART 6, 12 and 18 months prior to data collection respectively, and represented 86.4% of the sampling frame population of 9,693.

Table 1 shows the characteristics of the population on ART. Median age of the population at ART initiation was 36 years [IQR 30–43] and 64.6% were female. Over two thirds [69.0%], were enrolled into the ART program from voluntary counseling and testing [VCT] services, while 14.6% enrolled from PMTCT services and 4.9% from in-patient wards. The majority [63.6%] were classified as WHO stage I or II at the time of ART initiation, with only 2.4% missing WHO stage data at ART initiation. CD4+ counts at ART initiation were available for 84.9% of the population. Among those with available data, median CD4+ count was 206 cells/μL [IQR 137–286], and 37.0% had CD4+ count less than 200cells/μL at the time of ART initiation. Of note, the median CD4 count at ART initiation among adults who started ART as in-patients was 74 cells/μL [IQR 34–194] vs 218 cells/μL [IQR 150–288] among patients enrolled from VCT and 256 cells/μL [IQR 177–313] among patients enrolled from PMTCT clinics. The majority of the population initiated stavudine [58.9%] and zidovudine [40.9%] in the first-line regimen, all combined with lamivudine and nevirapine [90.3%] or efavirenz [9.1%].

**Table 1:** **Patient characteristics: overall distribution and by time on ART**

				Time on ART prior to data collection						
		Overall (all adults)		6 months on ART		12 months on ART		18 months on ART		***p-***value
		***n = 1,678***		***n = 644***		***n = 578***		***n = 456***		
		N	Weighted %	N	Weighted %	N	Weighted %	N	Weighted %	
***Patient-level characteristics***										
**Age groups**	18-25 years	200	10.7	61	8.4	76	12.1	63	12.1	
	26-35 years	619	35.5	229	33.8	210	34.8	180	38.4	
	36-45 years	562	33.8	227	36.4	185	32.4	150	32.2	
	46+ years	297	20.0	127	21.5	107	20.7	63	17.3	0.010
**Gender**	Female	1079	64.6	414	64.2	380	65.9	285	63.8	
	Male	229	35.4	229	35.8	195	34.1	170	36.2	0.361
**Source of referral to ART clinic**	VCT^a^	1157	69.0	460	70.6	400	68.4	297	67.7	
	PMTCT^b^	282	14.6	102	14.6	92	14.1	88	15.2	
	In-patient ward	70	4.9	21	4.0	20	4.1	29	6.9	
	Other^c^	169	11.5	61	10.8	66	13.4	42	10.2	0.172
**WHO stage at ART initiation** ^**d**^	Stage I and II	1019	63.6	448	71.7	320	60.5	251	56.8	
	Stage III	559	32.7	163	25.5	218	35.4	178	39.0	
	Stage IV	65	3.7	20	2.8	26	4.1	19	4.3	<.001
**CD4+ count (cells/μL) at ART initiation**	0 – 199	689	37.1	180	25.7	273	42.1	236	46.0	
	200 +	753	47.8	357	58.1	236	45.3	160	37.5	
	Missing	236	15.1	107	16.3	69	12.6	60	16.5	<.001
**Initial ART regimen containing…**	Stavudine	942	58.9	184	34.1	387	65.3	371	83.3	<.001
	Zidovudine	731	40.9	459	65.8	188	34.1	84	16.6	
	Tenofovir	5	0.2	1	0.1	3	0.6	1	0.1	
***Characteristics of clinic attended***										
**Location**	Rural	599	50.0	215	47.6	221	53.6	163	49.1	
	Urban	1079	50.0	429	52.4	357	46.4	293	50.9	0.781
**Facility level**	Health center	914	64.2	349	63.8	326	66	239	62.5	
	Hospital	764	35.8	295	36.2	252	34	217	37.5	0.928
**Facility ownership**	Faith-based	537	25.3	212	23.0	183	25.6	142	27.7	
	Public	1141	74.7	432	77.0	395	74.4	314	72.3	0.123
**Start of performance based financing**	2004-2006	919	55.2	363	55.7	332	59.0	224	50.1	
	2007-2008	759	44.9	281	44.3	246	41.0	232	49.9	0.628
**Home visits support for PLWHA** ^**e**^		887	50.9	333	49.6	304	52.6	250	50.6	0.942

^a^VCT = Voluntary Counseling and Testing services; ^b^PMTCT = Prevention of Mother-to-Child HIV Transmission.

^c^Includes Tuberculosis clinics [n = 17], outpatient services [n = 45] and other modes of admission [n = 107].

^d^Adults missing data on WHO stage at ART initiation [n = 35, 2.1%].

^e^PLWHA = Persons Living with HIV/AIDS.

Half the population attended health facilities located in rural areas [50.0%], and the majority attended health centers [64.2%, vs 35.8% at hospitals]; and public government facilities [74.7%, vs 25.3% at faith based health facilities]. All health facilities had performance based financing Nearly half [44.9%] of the population attended health facilities where performance based financing had been introduced more recently [2007–2008 vs. 2004–2006] and half attended health facilities where home visit support services for patients who miss health facility visits were available [50.9%].

When comparing the population by time of ART initiation, the population who initiated ART 6 and 12 months prior to data collection had a higher proportion of older adults [36 years and older], adults with WHO Stage I and II at ART initiation, and adults with CD4+ count above 200 cells/μL at ART initiation, than the population who initiated ART 18 months prior to data collection. Stavudine use in the initial ART regimen declined from 83.3% to 65.3% and 34.1% for adults who had initiated ART 18, 12 and 6 months previously, while zidovudine use increased from 16.6% to 34.1% and to 65.8% respectively.

Of the population that initiated ART, 5.5% transferred out to other health facilities. Of the remaining population, attrition was observed among 6.8%, with 2.9% documented to have died and 3.9% lost to follow-up [Table 2]. Attrition among adults who had initiated ART 6, 12 and 18 previously was 3.5%, 8.8% and 8.8% respectively. The population attrition rate was 7.5/100 PY, with deaths accounting for 3.2/100 PY and loss to follow-up 4.3/100PY [Table 2]. Attrition rates among adults who had initiated ART 6, 12 and 18 previously were 6.9/100 PY, 9.1/100 PY and 6.4/100 PY respectively.

**Table 2:** **Proportion and rate of attrition and transfers per 100 person years: overall and by time on ART**

				Time on ART prior to data collection								
	Overall (all adults)			6 months on ART			12 months on ART			18 months on ART		
	1509 person years			330 person years			559 person years			619 person years		
	1678 adults			644 adults			578 adults			456 adults		
Proportions:	n	Weighted %	95% CI	n	Weighted %	95% CI	n	Weighted %	95% CI	n	Weighted %	95% CI
Attrition	135	6.8	[6.0 – 7.8]	25	3.5	[2.7 – 4.5]	58	8.8	[7.1 – 10.9]	52	8.8	[7.9 – 11.4]
Recorded death	48	2.9	[2.4 – 3.5]	13	1.6	[1.1 – 2.3]	16	2.9	[2.0– 4.1]	19	4.6	[3.5 – 6.0]
Lost to follow-up	87	3.9	[3.4 – 4.5]	12	1.9	[1.3 – 2.7]	42	5.9	[5.0 – 7.0]	33	4.2	[3.2 – 5.6]
Transfer out	99	5.5	[4.5 – 6.8]	34	6.1	[4.5 – 8.2]	26	3.6	[2.5 – 5.3]	39	6.9	[4.8 – 9.8]
Retained on ART	1444	87.7	[86.0 – 89.2]	585	90.4	[88.4 – 92.2]	494	87.6	[84.7 – 90.0]	365	84.3	[80.4 – 87.5]
**Rates:**	**No.**	**Weighted rate**	**95% CI**	**No.**	**Weighted rate**	**95% CI**	**No.**	**Weighted rate**	**95% CI**	**No.**	**Weighted rate**	**95% CI**
Attrition	135	7.5	[6.1 – 9.3]	25	6.9	[4.3 – 11.9]	58	9.1	[6.6 – 12.7]	52	6.4	[4.6 – 9.2]
Recorded death	48	3.2	[2.3 – 4.5]	13	3.2	[1.7 – 6.9]	16	2.9	[1.6 – 5.6]	19	3.3	[2.0 – 5.9]
Lost to follow-up	87	4.3	[3.3 – 5.6]	12	3.7	[1.8 – 8.8]	42	6.1	[4.2 – 9.2]	33	3.1	[2.0 – 4.9]
Transfer out	99	6.0	[4.7 – 7.8]	34	12.1	[7.9 – 19.4]	26	3.7	[2.4 – 6.3]	39	5.0	[3.4 – 7.5]
Retained on ART	1444	95.7	[92.8 – 98.7]	585	179.5	[174.5 – 184.6]	494	90.3	[88.1 – 92.6]	365	61.1	[59.5 – 62.7]

Note: Some percentages may not add to 100% due to rounding.

Table 3 displays patient- and health facility-level characteristics associated with attrition in the bivariate and multi-variable analyses adjusting for various demographic, clinical and site characteristics. The adjusted hazard ratio [aHR] for attrition was highest for adults enrolled from in-patient wards: aHR 4.2 [95% CI: 3.0-5.7]; and PMTCT services: aHR 2.2 [95% CI: 1.6-3.0], compared to those enrolled from VCT services. Compared to adults who initiated ART 18 months earlier, adults who initiated ART 12 months earlier had higher hazard of attrition: aHR 1.8 [95% CI: 1.3-2.5]; but no significant difference was observed with adults who had initiated ART in the previous 6 months: aHR 1.3 [95% CI: 0.9 – 1.9]. A higher hazard of attrition was also observed among men: aHR 1.4 [95% CI: 1.0-1.8] than women; and adults attending urban health facilities: aHR 1.4 [95% CI: 1.1-1.8] compared to those attending rural facilities.

**Table 3:** **Crude and adjusted Cox proportional models for the hazard of attrition (death or loss to follow-up)**

		Bivariate analysis			Multi-variable model n = 1,678		
	Attrition N	HR of attrition	95% CI	p-value	aHR of attrition	95% CI	p-value
**Time on ART prior to data collection**							
18 months on ART	52	Ref			Ref		
6 months on ART	25	1.1	[0.8 – 1.7]	0.517	1.3	[0.9 - 1.9]	0.190
12 months on ART	58	1.7	[1.2 - 2.4]	0.003	1.8	[1.3 - 2.5]	0.000
**Age**							
18-25 years	27	Ref			Ref		
26-35 years	48	0.8	[0.5 - 1.2]	0.195	0.8	[0.5 - 1.3]	0.395
36-45 years	44	0.9	[0.6 - 1.2]	0.318	1.0	[0.7 - 1.4]	0.864
46+ years	16	0.6	[0.4 - 0.9]	0.014	0.8	[0.5 - 1.3]	0.379
**Gender**							
Females	48	Ref			Ref		
Males	87	1.3	[1.0 - 1.6]	0.066	1.4	[1.0 - 1.8]	0.027
**Source of referral to ART clinic**							
Voluntary Counseling and Testing	70	Ref			Ref		
PMTCT services	35	2.1	[1.6 - 2.7]	0.000	2.2	[1.6 - 3.0]	0.000
In-patient ward	15	4.1	[3.0 - 5.6]	0.000	4.2	[3.0 - 5.7]	0.000
Other	15	1.6	[1.2 - 2.2]	0.001	1.5	[1.1 - 2.0]	0.004
**CD4+ count at ART initiation (cells/μL)**							
0 – 199	69	Ref			Ref		
200+	45	0.7	[0.5 - 0.9]	0.005	0.8	[0.6 - 1.0]	0.031
Missing	21	1.0	[0.7 - 1.3]	0.763	1.0	[0.8 - 1.3]	0.811
**Initial ART regimen**							
Stavudine containing	80	Ref			Ref		
Zidovudine containing	54	1.1	[0.9 - 1.3]	0.423	1.0	[0.8 - 1.2]	0.944
Tenofovir containing	1	1.6	[0.7 - 3.6]	0.240	1.7	[0.8 - 3.9]	0.165
**Location**							
Rural	32	Ref			Ref		
Urban	103	1.5	[1.1 - 2.0]	0.005	1.4	[1.1 - 1.8]	0.013
Facility level*							
Hospitals	52	Ref			Ref		
Health centers	83	0.9	[0.7 - 1.2]	0.483	0.8	[0.6 - 1.1]	0.226
**Start of performance based financing**							
2007-2008	85	Ref			Ref		
2004-2006	50	0.9	[0.6 - 1.2]	0.239	0.8	[0.6 - 0.9]	0.016
**Home support visits for PLWHA available**							
No	60	Ref			Ref		
Yes	75	1.2	[0.9 - 1.6]	0.415	1.2	[1.0 - 1.6]	0.097

*Facility ownership variable (public vs faith based) excluded from the model due to collinearity with facility level variable.

The hazard of attrition was lower among adults whose CD4+ count at ART initiation was 200 cells/μL or greater, aHR 0.8 [95% CI: 0.6-1.0], compared to those who initiated ART with CD4+ counts below 200 cells/μL. Lower attrition hazard was also observed among adults who attended health facilities that had longer-standing performance-based financing [since 2004–2006], aHR 0.8 [95% CI: 0.6-0.9], compared to patients attending health facilities where performance based financing had began more recently [2007–2008]. Age, initial ART regimen, level of health facility and availability of home visit services were not significantly associated with attrition in adjusted analyses.

## Discussion

We assessed levels of attrition 6, 12 and 18 months following ART initiation among a nationally representative sample of adults receiving HIV services in Rwanda. We estimated overall population attrition to be 6.8% and the attrition rate as 7.5/100 PY. Lowrance et al., who explored this question in the early phase of the Rwanda ART program [2004–2005], reported attrition of 6.7% of patients at 6 months and 9.5% at 12 months on ART [13]. We found much lower attrition at 6 months, 3.5%, and approximately the same attrition at 12 months, 8.8%, in 2007–2008, suggesting a significant reduction in attrition at the 6 months following ART initiation. Our results tally with more recent findings using a significantly larger sample in two regions of Rwanda [17]. This is an important finding because the risk of attrition tends to be highest immediately after ART initiation, and is usually due to high mortality within this time period [18–20]. These findings may reflect the benefits of initiating ART earlier in HIV disease, as the median CD4+ count at ART initiation in our sample was 65 cells/μL, higher than that observed by Lowrance et al.

While Lowrance et al. observed that death and LTF contributed equally to attrition in the early phase of ART scale-up in Rwanda, our data indicate that LTF increasingly accounts for the majority of attrition. This may reflect deteriorating ascertainment and documentation of deaths as the ART program expands, and an increase in undocumented transfers as ART services are decentralized [20–22]. Stronger linkages and feedback between communities and health facilities [to report deaths] and between health facilities [to report transfers] could result in more accurate estimations of true loss to follow-up, mortality, and transfers and thus more complete assessment of the ART program impact.

While the level of attrition observed in our sample was in-line with other findings from Rwanda [13, 14, 23, 17], it is substantially lower than that observed in other programs within the East African region, which report attrition in the range of 18% to 40% 12 months after ART initiation [24–28]. Multiple factors may be contributing to the low attrition in ART programs in Rwanda. First, the median CD4+ count at ART initiation in our population was significantly higher than has been observed in other countries [18, 25, 29, 30], and could account for this association. Our results also showed that CD4+ counts >200/μL were associated with a 20% reduction in attrition. Notably, patients whose CD4+ counts at ART initiation were missing had similar hazard of attrition to the patients with the lowest CD4+ counts. This finding suggests that patients whose CD4+ counts are not assessed and recorded at ART initiation may be as vulnerable to attrition as the sickest patients. Secondly, adults in the Rwanda ART program tend to have high adherence to ART [31, 32] which in turn can reduce mortality, and thus attrition. This is further evidenced by adherence and viral load assessments conducted on a subset of participants in this analysis which revealed that overall [combining adults on ART 6, 12 and 18 months], 94% and 78% took 100% of their medication in the 3 and 30 days preceding the interview, and 83% had viral load under 40 copies/mL at the time of interview [15].

The most significant factor associated with increased hazard of attrition in our study was starting ART while admitted within an in-patient ward. This finding has been observed by others [33], and is likely due to high mortality in this severely ill group and LTF due to undocumented deaths [20, 34]. Indeed, the median CD4 count at ART initiation among in-patients was about a third of observed among other study participants. Recent recommendations by WHO and the Rwanda Ministry of Health [35–37], which endorse ART initiation at higher CD4 counts [350 cells/μ/L and more recently 500 cells/μ/L], could result in adults initiating ART earlier in HIV disease, and thus lower attrition. Enrolling from PMTCT services was also associated with high attrition. This has been observed in a number of African programs [38, 39], and is a challenge worth addressing as countries begin to implement for life-long ART for all pregnant and breastfeeding women. The finding that men had higher attrition than women is not uncommon in African HIV treatment programs [40–44]; this is a high risk group that requires targeted interventions to minimize attrition.

Adults who attended health facilities with a longer duration of performance based financing had a lower hazard attrition in our analysis. Although our analysis lacked data to comparatively examine attrition at facilitates without performance based financing, implementation of performance based financing is perceived as a strong motivator for provision of better health care services [45–47], and may have contributed to improvements in patient outcomes at these health facilities clinics. It is possible that sites that initiated performance based financing earlier had other unmeasured characteristics that influenced attrition outcomes, and further research should examine the specific impact of this strategy on lowering attrition in African ART programs.

Our study did not find a correlation between home visits and attrition, a finding in line with results of a multi-country ecological study examining the impact of site-level factors on attrition [48]. Whereas it has been observed that home-visits can be associated with better retention [49], it is likely that in our setting of high retention, the impact of home visits was minimal, particularly if targeted to patients with high risk of attrition that may have insurmountable barriers to returning to care.

Our study had a number of strengths. We had a large sample of adults drawn from a nationally representative sample of adults on ART, and there was representation from different types of health facilities in various settings. The study was conducted in one of the first few African countries to achieve universal ART access. In multivariate analyses, we explored associations between both patient- and health facility- level factors and attrition. A limitation to note, however, is that the study lacked sufficient power to examine factors associated with death and LTF in separate models or present results stratified by time on ART. Despite this, the results effectively demonstrate the low attrition rates and some key factors associated with the risk of attrition for the overall population.

## Conclusion

Our results suggest sustained low attrition in the Rwanda national ART program in the context of significant expansion in the number of clinics providing HIV care and treatment services and the number of patients on ART. Loss to follow-up was the more significant contributor to attrition suggesting that better ascertainment and documentation of deaths and patient transfers may be necessary. In this context of universal ART access, specific groups remain at risk of high attrition [including in-patients, men, PMTCT clients and adults attending urban facilities] and require targeted interventions to further reduce attrition. Performance-based financing could be a useful tool in minimizing attrition in this context.
